# Estimating Fast Neural Input Using Anatomical and Functional Connectivity

**DOI:** 10.3389/fncir.2016.00099

**Published:** 2016-12-20

**Authors:** David Eriksson

**Affiliations:** ^1^Center for Neuroscience, Albert Ludwig University of FreiburgFreiburg, Germany; ^2^BrainLinks-BrainTools, Albert Ludwig University of FreiburgFreiburg, Germany

**Keywords:** anatomical connectivity, functional connectivity, perturbation, contextual signaling, neural circuits

## Abstract

In the last 20 years there has been an increased interest in estimating signals that are sent between neurons and brain areas. During this time many new methods have appeared for measuring those signals. Here we review a wide range of methods for which connected neurons can be identified anatomically, by tracing axons that run between the cells, or functionally, by detecting if the activity of two neurons are correlated with a short lag. The signals that are sent between the neurons are represented by the activity in the neurons that are connected to the target population or by the activity at the corresponding synapses. The different methods not only differ in the accuracy of the signal measurement but they also differ in the type of signal being measured. For example, unselective recording of all neurons in the source population encompasses more indirect pathways to the target population than if one selectively record from the neurons that project to the target population. Infact, this degree of selectivity is similar to that of optogenetic perturbations; one can perturb selectively or unselectively. Thus it becomes possible to match a given signal measurement method with a signal perturbation method, something that allows for an exact input control to any neuronal population.

## Introduction

**Figure 1:**
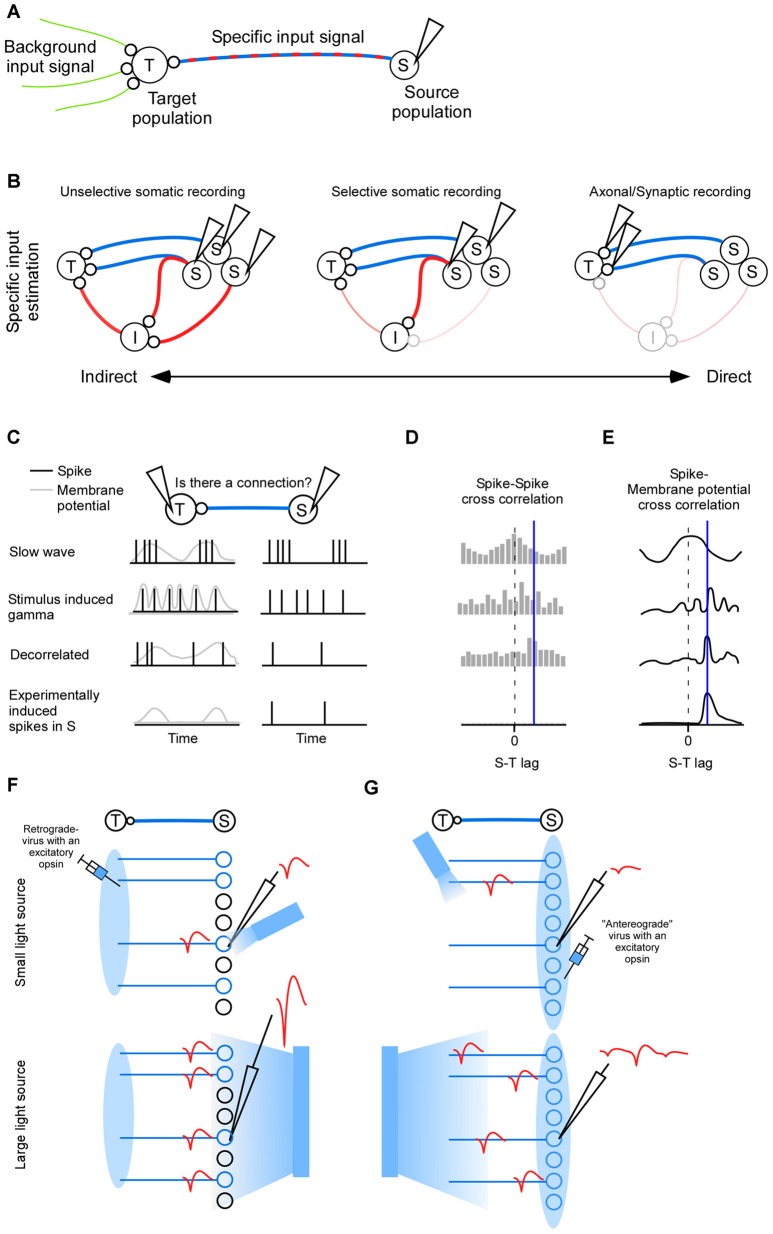
**Inputs to population (T). (A)** The complete input to a neuronal population (T) can be divided into a background input and a specific input (S).** (B)** Indirect to direct spectrum of inter-cellular signaling. The indirect route goes via indirect neurons (I). *Left*: activity is recorded in neurons (S) that have a polysynaptic path to the target (T). *Middle*: activity is recorded in neurons that have a direct connection to (T). Some of those neurons may also send collaterals elsewhere, hence contributing to the indirect activity. *Right*: synapse specific recordings allows quantification of the direct input to the target neuron exclusively while sparing indirect paths. **(C)** Functional connectivity between (S) and (T) is crucially dependent on the type of neuronal activity to which the connectivity measure is applied (rows). Correlated activity in terms of slow wave sleep generates a strong spike incidence at zero lag (first row), correlated activity in terms of high frequency gamma oscillation generates a spike incidence with a periodicity (second row), decorrelated activity is more likely to give a high spike incidence at the lag defined by the connection (third row), and experimentally induced single pre synaptic spikes are more likely to show a spike incidence only at the lag defined by the connection (fourth row). Postsynaptic activity is either spikes (black) or membrane potential (gray). **(D)** Hypothetical cross-correlations between (S) and (T) for the different spiking activity types shown in **(C)**. The delay of the connection is indicated by a blue vertical line. **(E)** Hypothetical cross-correlations between (S) and (T) for the different spike-membrane potential activity types shown in panel **(C)**. **(F)** For somatic phototagging the light should be small and directed towards the electrode tip (top). The larger the emitter is the larger the population spike (red) will be (bottom). **(G)** For axonal phototagging a small light source may miss the axon of the recorded neuron. The recorded action potential will therefore be of low amplitude (top). Instead the light source may be large and positioned somewhere in the target area (bottom). Although many neurons will be activated the axonal conduction velocity heterogeneity separates the spikes in time.

Ideally the neuroscientist ought to understand how all the inputs to a population affect its output activity (Jonas and Kording, 2016). A pragmatic version of this goal is to compare the importance of one specific input (S), to all remaining inputs (B) in generating the output activity in population (T; Figure 1A). The background input (B) can potentially be estimated using optogenetic inhibition (Eriksson, 2016). Here we will review methods for estimating the complementary specific input signal which originates from the source population (S).

Since the specific signal governs the activity in the target population it might be tempting to estimate the specific signal by inhibiting it and measuring how the target activity changes. The resulting change may have very little to do with the specific signal (Lien and Scanziani, 2013). To illustrate this one can imagine that the specific signal conveys a simple trigger that starts a complex computation in the target population. When the specific signal is inhibited the activity in the target population is radically simplified and one would falsely conclude that the specific signal is a complex signal. To be able to detect such non-linear effects it is crucial to measure the specific signal directly.

In the first two sections we review mathematical and anatomical approaches for identifying projecting neurons. Their activity represent the specific signal. The first section deals with mathematically oriented methods which typically identifies both direct and indirectly connected neurons (Figure 1B left). In the second section we review experimentally oriented methods for identifying directly connected neurons primarily, although some of the identified neurons will inevitably send collaterals to indirect targets (Figure 1B middle). In the last section we review imaging methods for measuring the specific signal directly at the synapse (Figure 1B right).

## Unselective Recording

The experimentally least demanding method for approximating the unspecific direct and indirect signal that is running from the source to the target population is to insert one extracellular electrode array in each population. Linear and non-linear mapping methods can then be used to identify source units that convey information about the activity of the target units (Aggarwal et al., 2009; Graf et al., 2011; Aggarwal et al., 2013; Haxby et al., 2014; Kaufman et al., 2014). A problem with mapping methods is that although the source units convey information about the target units, this may not be because they send information to the target units, but because they receive information from them. Therefore such methods are suitable to apply for pathways with a large delay such that the lag between source and target can be used to infer causality. Granger causality partially solves this problem since it takes the (causal) history into account. It requires relatively little data, and is typically used for linear interactions. To deal with nonlinear interactions, the more data intensive method called transfer entropy is applied (Vicente et al., 2011). To control for the influences of a third area (the common source problem) one can condition the interaction estimation on recordings done in additional areas (Bastos et al., 2015). Even non-simultaneous recordings in overlapping areas can be “stitched” together to provide a more complete description of the interaction (Soudry et al., 2013; Turaga et al., 2013). Finally if one has the luxury to choose from a few well defined and constrained models, one can apply dynamic causal modeling to identify which of those models best describe the interaction between the source and the target population (Pinotsis et al., 2012; Friston et al., 2013; Kobayashi and Kitano, 2013; Roudi et al., 2014).

**Figure 2:**
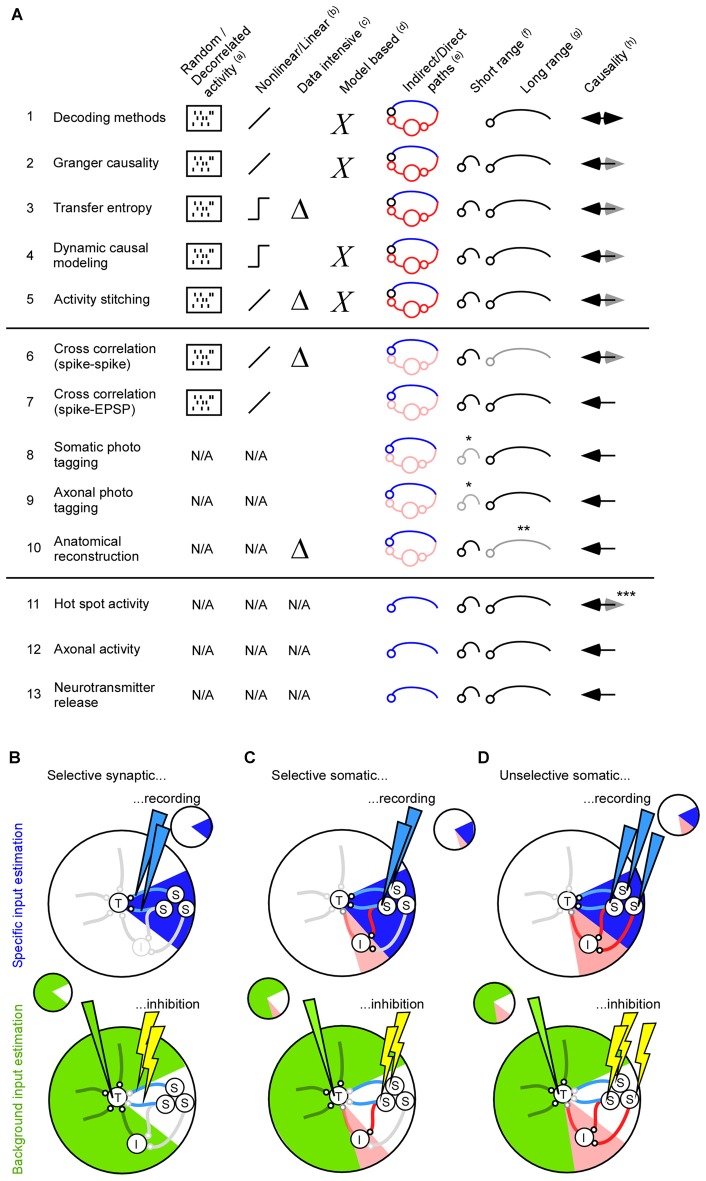
**Methods for finding direct and indirect pathways. (A)** Summary of 13 methods for estimating inter-cellular signals. To measure the inter-cellular signals one needs to identify the projecting neuron (1–10), or connecting synapse (11–13).** (a)** For the functional methods connected neurons are found most reliable if the neuronal activity in both the source and the target area is decorrelated (1–7). For the anatomical methods, such as photo tagging and neurite reconstructions, the neuronal activity is not used and, as such, is not a limiting factor (8–13). **(b)** The functional methods can be divided into those that extract linear relationships between the source and the target population, and those that extract non-linear relationships. **(c)** Transfer entropy extracts non-linear relationships and is therefore data intensive. **(d)** Dynamic causal modeling relies on modeling assumptions. **(e)** Methods that identify projection neurons with mathematical means will typically result in a large number of indirectly connected neurons. **(f,g)** The suitability for finding short and/or long range connectivity. Somatic- and axonal photo tagging of short range connections within 200–500 micro-meter is limited by virus diffusion (*). Anatomical reconstruction of long-range axons using electronmicroscopy is extremely resource intensive (**). **(h)** Decoding methods can only give causal information if the connection between source and target is directed and having a long delay. Anatomical based methods (8–10) and those that extract the activity in the synapse (12–13) can most reliably identify causal/projecting neurons. Calcium hot-spot derived post synaptic activity may be influenced by back propagating action potentials and is therefore less suited for identifying causal activity (***). **(B)** The total input to a neuron from fast and slow chemical synapses, astrocytes, vasculatures, extracellular ions, ephaptic signals and gap junctions can be divided into a specific signal (blue, top) and a background signal (green, bottom). In this review article, we have focused on how to estimate the specific input from fast chemical synapses, gap junctions and ephaptic effects. The background input can be addressed by inhibiting the specific signal. Since optogenetic inhibition has a faster onset than the feedback time of astrocytes, vasculature, slow chemical synapses, and the responses of extracellular ions, optogenetic inhibition can be used to estimate their input contribution (Eriksson, 2016). To cover all inputs to the neuron a rough guideline is to inhibit and record the same signal. For example, if selective synaptic/axonal inhibition is used for estimating the background input, in which only direct pathways will be affected, it is preferable to estimate signal (S) using the synaptic activity based methods (A11–A13). **(C)** If selective somatic inhibition is used for estimating the background input, in which relatively few indirect pathways will be affected, it is preferable to estimate signal (S) using selective somatic recordings (A6–A10). **(D)** If unselective somatic inhibition is used for estimating the background input, in which many indirect pathways will be affected, it is preferable to estimate signal (S) such that the effect of indirect pathways can be estimated (A1–A5).

For short range interactions the local field potential (LFP) may be an additional unspecific factor that influences the activity in the target population. The extracellular electric fields generated by neuronal activity are strong enough to modulate membrane potentials and spiking probabilities (Fröhlich and McCormick, 2010; Anastassiou et al., 2011). To quantify the relation between the spiking activity and the extracellular electrical field one can average the LFP across the spikes (Nauhaus et al., 2009; Rasch et al., 2009). A perfect match between the spike and LFP is not expected, though, since the LFP is the combined result of neurons and glia (Anastassiou and Koch, 2014). Nevertheless, LFP frequencies below 15 Hz are the easiest to predict (Nauhaus et al., 2009; Rasch et al., 2009). This fits well with the fact that spike entrainment is particularly effective for ephaptic field frequencies below 8 Hz (Anastassiou et al., 2011). The predicted LFP components give information about how the membrane potential and spiking probability is modulated (Anastassiou et al., 2010; Okun et al., 2010; Haider et al., 2016). Since the LFP changes across different cortical layers, and since neurons are sensitive to those spatial changes, the LFP should preferably be recorded using a laminar electrode (Anastassiou et al., 2010; Linden et al., 2011). To summarize, both individual neurons and ephaptic effects can contribute to the unselective signaling between two neuronal populations. The reviewed mathematical methods can be used to identify which neurons are important, and/or whether ephaptic effects should be taken into account, for understanding the target activity (see Figures 2A1–5).

## Selective Somatic Recording

Here we review functional and anatomical methods to find neurons that directly connect to a certain population of neurons (see Figures 2A6–10). Once those neurons have been identified, their activity can be used to infer the inter-cellular signal.

### Functional Techniques

We will focus on cross-correlations between the pre- and postsynaptic neurons for estimating neuronal connectivity (Perkel et al., 1967; Ts’o et al., 1986; Fujisawa et al., 2008; Berényi et al., 2014). With the introduction of multi-channel extracellular recordings those methods have been used to estimate short and long range connectivity (Berényi et al., 2014), feedforward connectivity from primary visual cortex to secondary visual cortex (Zandvakili and Kohn, 2015) and local connectivity (Isomura et al., 2009). Although general correlations in neuronal data can be tested for significance using powerful mathematical methods (Grun, 2009), we argue here that it is crucial to acquire data that is suitable for applying cross-correlation techniques. We will cover endogenous/spontaneous activity caused by the brain itself, and exogenous activity caused by the experimenter.

Cross-correlations have a limitation whereby detected relationships may not correspond to real anatomical connections. For example, a third brain area targeting the neuronal pair of interest could generate spurious connections (i.e., the common source problem). Importantly, the number of spurious connections is dictated by the brain state (Figure 1C). For slow wave sleep the activity of different neurons co-vary with zero-lag (first row in Figures 1C–E). Close to 100% of those apparent connections will be false positives because they are not anatomically connected. For a more decorrelated (or random) spontaneous activity, a more reasonable estimate of the connection probability of 0.3%–0.5% is obtained for spiking activity *in vivo* (Fujisawa et al., 2008; Zandvakili and Kohn, 2015). Using *in vitro* patching a larger connectivity probability of 2% is seen between pyramidal cells which may be explained by the more sensitive post synaptic potential (Nowak et al., 1999; Holmgren et al., 2003; Song et al., 2005; Fujisawa et al., 2008). Even during the more decorrelated state typically associated with sensory stimulation there are detectable correlations between neurons that are not necessarily connected in the anesthetized animal (Yu and Ferster, 2010), and in the awake animal (Fries et al., 2001; Ray and Maunsell, 2010; second row in Figure 1C). Therefore, although the brain automatically randomize/decorrelated activity by means of heterogenous populations of neurons and inhibitory neurons (Padmanabhan and Urban, 2010; Renart et al., 2010; Tetzlaff et al., 2012; Bernacchia and Wang, 2013), in some cases it may be advantageous to artificially decorrelate neurons (third row in Figure 1C). Decorrelation has previously been accomplished by optogenetically injecting a one-dimensional noise signal (Han and Boyden, 2007). In the future, the degree of decorrelation might be enhanced using various light-sculpting approaches (Rickgauer and Tank, 2009; Dal Maschio et al., 2010; Zahid et al., 2010; Katona et al., 2012; Quirin et al., 2013; Schrödel et al., 2013; Rickgauer et al., 2014). Even single neurons can be selectively activated by the experimenter (fourth row in Figure 1C; Rickgauer et al., 2014; Szabo et al., 2014; Packer et al., 2015).

For estimating connectivity, the background input to a neuron is both beneficial and problematic. The background input creates spurious connections and adds variability to the connectivity estimation. On the other hand, this input may be crucial for the generation of action potentials; thus, without this input it would be impossible to detect a connection using extracellular recordings or calcium imaging. One alternative is to provide this additional input via artificial stimulation. The firing threshold can be decreased using two-photon stimulation of a single postsynaptic neuron (Prakash et al., 2012). A small number of postsynaptic neurons can now be activated, and even decorrelated, in similar ways using light patterning methods (see references above). The sparse activation practically eliminates the problem of common source input. Also sparse activation of presynaptic neurons may be beneficial when studying weak long range connections. To this end, projection neurons may be selectively stimulated through retrograde labeling (Wickersham et al., 2007a,b; Reardon et al., 2016). Overall, it may be pragmatic to try to measure connectivity in terms of postsynaptic spikes, since spikes are reliably detected using two-photon imaging of calcium indicators or with dense extracellular recordings, something which is not yet established with voltage indicators *in vivo*.

Ultimately, connectivity should be estimated in terms of the postsynaptic potential (Figure 1E). Ongoing attempts combine whole-cell recordings with selective two-photon stimulation of potential presynaptic cells (Packer et al., 2012). The yield for these whole-cell recordings may be increased through the use of patching robots, which may allow for the simultaneous patching of multiple neurons (Kodandaramaiah et al., 2012). Furthermore, fluorescent voltage markers might allow for the recording of membrane potentials across multiple neurons via two-photon imaging (Akemann et al., 2012; Knopfel, 2012; Flytzanis et al., 2014; St-Pierre et al., 2014; Vogt, 2014; Yang and St-Pierre, 2016).

### Anatomical Techniques

Neurons that project to a specific target area can be found by anatomical means. To this end a retrogradely transported virus expressing an excitatory opsin is injected in the target area (Zhang et al., 2013; Figure 1F), or a specific cell type is targeted using transgenic animals (Lima et al., 2009). A brief light pulse will then evoke a spike in expressing neurons (Lima et al., 2009). If a spontaneously evoked spike matches this light evoked spike waveform then it is assumed that it was generated by the expressing neuron. The problem is that multiple expressing neurons will fire simultaneously to the brief light pulse such that spike sorting becomes difficult. Even neurons far from the electrode may show up in the population spike, since the number of neurons increases with distance (Du et al., 2011). Therefore one should use a small optical fiber to illuminate as small a volume as possible (Stark et al., 2012, 2014; Pi et al., 2013; Wu et al., 2015). Indeed, the required emitting light power for evoking a spike can be reduced by several orders of magnitude if the emitter is decreased in size, indicating a large increase in selectivity (Buzsáki et al., 2015), and somatic stimulation (Wu et al., 2015). Indirectly activated neurons can be detected by means of the spike jitter since it will in general be larger for an indirect activation than for a direct activation (Zhang et al., 2013). Synaptic antagonists can be used to block indirect activation (Lima et al., 2009; Zhang et al., 2013). To avoid the population spike photo-tagging can be done with inhibition instead of excitation (Courtin et al., 2014). Here the latency until spike cancelation is indicative of an indirect or direct inhibition. In addition the Becquerel effect can be subtracted since it will not be time-locked to the spontaneous spikes.

Projection neurons can also be found by infecting the source area with an excitatory opsin and by evoking an anti-dromic spike in the projecting neurons by illuminating the axonal terminals (Sato et al., 2014; Li et al., 2015; Figure 1G). The fundaments for this technique were laid out several decades ago when researches started to use anti-dromic electric stimulation of axons (Miller, 1975; Cleland et al., 1976; Lipski, 1981; Ferster and Lindström, 1983). Although, electrical stimulation is simpler than optogenetic stimulation it may require comparable higher stimulation intensities since the electric field decays quicker over space than the photon distribution. Indeed, in a beautiful study of geniculo-cortical connectivity it was noted that the electrical stimulation had to be so strong that it sometimes leads to small lesions (Ferster and Lindström, 1983). Typical light intensities may at worst cause reversible changes in neuronal activity (Stujenske et al., 2015). In comparison to the retrograde approach in which a virus is taken up by the presynaptic terminals, the axonal stimulation approach may run the risk of stimulating en passant axons. Furthermore, it may be difficult to know where the emitter should be placed given the location of the recorded neuron (in the retrograde approach the emitter should be placed where the neuron/electrode is; Figure 1G). Instead it might be advantageous if the emitter is very large such that many axons are stimulated. Note that the population-spike is weaker for axonal stimulation since the relatively large heterogeneity of axonal conduction delays separates the evoked spikes in time. Axonal phototagging also has the advantage that the number of target structures is not constrained by the number of opsins with non-overlapping wavelengths (e.g., blue and red depolarizing opsins Yizhar et al., 2011; Lin et al., 2013; Klapoetke et al., 2014; Emiliani et al., 2015), as is the case for the retrogradely transported opsin approach. To assure the identity of the sorted unit one can do a collision test (Ciocchi et al., 2015; Li et al., 2015), and to control for collaterals one can assure a low spike jitter, and/or apply synaptic blockers (Sato et al., 2014). Although the choice between somatic or axonal phototagging depends on the question at hand, there is so far no study that has systematically studied the advantages and disadvantages of those two approaches.

It is possible to approximate neuronal connectivity based on axonal and dendritic reconstructions (Stepanyants and Chklovskii, 2005). Typically, the distance between neurites indicates whether there is a synapse. Similarly to the functional approaches discussed above, this anatomical approach may produce both false negatives and spurious connectivity (Stepanyants and Chklovskii, 2005). Dense extracellular recordings may allow the position of a recorded cell group to be estimated and matched to histology (Blanche et al., 2005; Scholvin et al., 2016). Various tissue-clearing approaches may increase the chance of finding a match between an extracellularly recorded cell and a histologically-identified cell, since the brain remains intact and therefore is minimally distorted (Chung et al., 2013; Ke et al., 2013; Miyawaki, 2015). Finally, in one intriguing study, electron microscopy was used to reveal reconstructed connections in a 350 μm × 450 μm × 52 μm block of tissue, combined with two-photon calcium imaging of the corresponding tissue (Bock et al., 2011). If done properly the electron microscopy reconstruction will generate a negligible number of spurious or false negative connections (Denk and Horstmann, 2004; Jurrus et al., 2009). Recent developments could facilitate reconstruction within a larger volume, if not the entire mouse brain (Hua et al., 2015; Mikula and Denk, 2015).

## Axonal/Synaptic Recording

Since the projection signal can be seen as synaptic activity, another approach is to measure the activity in and around the synapse (see Figures 2A11–13). The post-synaptic activity gives a localized activity in terms of a hot-spot (Jia et al., 2010; Chen et al., 2011). Although this activity is related to the synaptic activity it is also dependent on the postsynaptic activity such as back propagating action potentials (Jia et al., 2010). Calcium activity in the axonal terminal is much less influenced by the postsynaptic activity (Andermann et al., 2013; Gunaydin et al., 2014). It is even possible to target individual axon terminals with two-photon axonal calcium imaging (Cruz-Martin et al., 2014). Finally, to address synaptic depression and facilitation (Markram and Tsodyks, 1996; Tsodyks and Markram, 1997), it might be optimal to measure the neuro transmitter release (Schulze et al., 1999; Nguyen et al., 2010). Recent, fluorescent markers for glutamate showed both cellular (synaptic) and millisecond resolution (Marvin et al., 2013). A future possibility is to measure the neurotransmitter in identified synaptic clefts by means of a genetically encoded presynaptic fluorescent marker and a genetically encoded postsynaptic transmitter marker (Lin and Schnitzer, 2016).

## Conclusion

Here we have reviewed ways to estimate the signal that runs from one neuronal population to another. Some of the methods are suitable to estimate the combined contribution from mono- and poly-synaptic signals that run along direct and indirect pathways, whereas other methods can be used to selectively target the direct mono-synaptic signal between the two populations. This wide range of methods allow the researcher to tailor his/her experiment to the question at hand. In particular, if one wants to inhibit and record a specific input, one can tailor the input recording method to match the inhibition method (Figures 2B–D). If we inhibit and record the same input we will have an excellent control of the input to the target population.

## Author Contributions

The author conceived and performed the study.

## Funding

The article processing charge was funded by the German Research Foundation (DFG) and the University of Freiburg in the funding programme Open Access Publishing.

## Conflict of Interest Statement

The author declares that the research was conducted in the absence of any commercial or financial relationships that could be construed as a potential conflict of interest.
